# PediMS: A Pediatric Multiple Sclerosis Lesion Segmentation Dataset

**DOI:** 10.1038/s41597-025-05346-5

**Published:** 2025-07-10

**Authors:** Maria Popa, Gabriela Adriana Vișa, Ciprian Radu Șofariu

**Affiliations:** 1 Babeș-Bolyai University, Faculty of Mathematics and Computer Science, Department of Computer Science, Mihail Kogălniceanu 1, Cluj-Napoca, Romania; 2 The Clinical Pediatric Hospital Sibiu, Pompeiu Onofreiu 2-4, Sibiu, Romania

**Keywords:** Multiple sclerosis, Paediatric research

## Abstract

Multiple Sclerosis (MS) is a chronic autoimmune disease that primarily affects the central nervous system and is predominantly diagnosed in adults, making pediatric cases rare and underrepresented in medical research. This paper introduces the first publicly available MRI dataset specifically dedicated to pediatric multiple sclerosis lesion segmentation. The dataset comprises longitudinal MRI scans from 9 pediatric patients, each with between one and six timepoints, with a total of 28 MRI scans. It includes T1-weighted (MPRAGE), T2-weighted, and FLAIR sequences. Additionally, it provides clinical data and initial symptoms for each patient, offering valuable insights into disease progression. Lesion segmentation was performed by senior experts, ensuring high-quality annotations. To demonstrate the dataset’s reliability and utility, we evaluated two deep learning models, achieving competitive segmentation performance. This dataset aims to advance research in pediatric MS, improve lesion segmentation models, and contribute to federated learning approaches.

## Background & Summary

Multiple Sclerosis (MS) is a chronic neuroinflammatory and neurodegenerative disease of the central nervous system that primarily affects young adults between the ages of 20 and 50, with an estimated 2.5 million people affected worldwide^1^. However, pediatric cases are rare, accounting for approximately 5% of all MS cases^2,3^. According to the MS Atlas (https://www.atlasofms.org/map/global/epidemiology/number-of-children-with-ms), at least 30,000 children worldwide have been diagnosed with MS across 55 reported countries, with 40 cases documented in Romania.

Early detection and monitoring of MS progression are crucial and require repeated Magnetic Resonance Imaging (MRI) screenings^4,5^, The characteristics of MS lesions on MRI include an ovoid appearance with a typical distribution in the periventricular region, corpus callosum, centrum semiovale, brainstem, cerebellum, spinal cord, deep white matter structures, and basal ganglia. MS brain lesions appear hyperintense on T2-weighted and proton density (PD) studies and hypointense on T1-weighted images. Gadolinium-enhancing lesions on T1-weighted MRI are associated with new or newly active plaques^6^.

Over time, the clinical progression of patients with MS can be either progressive or relapsing-remitting (RRMS). The most common form of MS, relapsing-remitting multiple sclerosis (RRMS), is characterized by alternating periods of relapse and remission, during which lesions and symptoms may temporarily subside^7^. Longitudinal MRI screenings play a vital role in diagnosis and tracking disease progression by identifying and analyzing new or evolving lesions. However, manual lesion segmentation is a labor-intensive process, requiring significant expertise and time^8^, highlighting the need for automated solutions.

To address this challenge, researchers have developed various models for automatic MS lesion detection. However, the performance of these models is often constrained by the limited availability of diverse datasets, particularly for underrepresented populations such as pediatric patients^9^. Several international competitions have been organized to advance the field, including the MICCAI challenges in 2008 and 2016^4,10^, as well as the International Symposium on Biomedical Imaging (ISBI) challenge in 2015^11^. More recently, in 2021, MICCAI introduced another MS segmentation challenge focusing on longitudinal screenings^12^, followed by a new competition in 2024^13^. These challenges have contributed to the release of publicly available MS datasets. Apart from competition datasets, two additional open-access MS datasets exist: 3D-MR-MS^14^ and long-MR-MS^15^. However, all publicly available datasets exclusively feature adult MS patients, and to the best of our knowledge, there is currently no publicly available pediatric MS lesion segmentation dataset.

Pediatric MS differs significantly from adult MS in both clinical presentation and lesion characteristics. Children experience more frequent relapses due to heightened immune system activity and hormonal fluctuations. However, their progression to mild or severe disability is typically slower compared to adults^16,17^. Pediatric MS often follows an early-onset relapsing-remitting course, and MRI studies play a crucial role in characterizing lesion structure, progression, and recovery. Lesions in pediatric MS are more frequently found in the cerebellum and brainstem^16–19^ than in adult patients, which may impair age-expected cerebellar growth. Additionally, pediatric MS lesions tend to be less destructive and may exhibit a greater capacity for repair, as evidenced by the gradual recovery of T1 intensity over time^16,18^.

To develop more generalizable MS lesion segmentation models, enhance federated learning approaches, and improve clinical applicability, there is a need for datasets that capture a broader range of patient demographics and disease progressions. This study introduces a novel dataset with the following key contributions:

- **PediMS**^20^: The first publicly available MRI dataset specifically dedicated to pediatric multiple sclerosis lesion segmentation, containing diverse forms of MS.
- **Comprehensive Clinical Metadata:** The dataset includes patient demographic and clinical data, curated by medical experts.
- **Assess dataset quality:** We assess the dataset’s quality and utility by performing inference with a state-of-the-art deep learning model, trained on an existing MS dataset. The results provide insights into how well current models generalize to pediatric cases.

PediMS^20^ contains pediatric cases that exhibit greater inflammatory activity, including: higher lesion burden at onset compared to adults and larger lesion sizes, which can influence the performance of segmentation algorithms. While the dataset primarily supports lesion segmentation, it also included relevant clinical metadata, which could be leveraged in future research exploring other approaches. By making this dataset publicly available, we aim to support future research in MS lesion segmentation, facilitate the development of more robust AI-driven diagnostic tools, and encourage collaborative efforts in federated learning for pediatric neuroimaging.

## Material & Methods

We first collected data from 9 pediatric patients, each with between 1 and 6 time points, at the Sibiu Pediatric Clinical Hospital, Romania. Data collection and use were approved by the hospital’s Ethics Committee (approval no. ICR 3161/05.05.2025). Written informed consent for data sharing and future research use was obtained from the patients’ parents or legal guardians. The patients demographic data can be seen in Table 1.

**Table 1:** Patient demographic, where RR - relapse-remit.

Gender	Age onset	MS type
2 male	14 and 15 years	RR
7 female	6 to 17 years	RR

In general the onset symptoms were numbness, vision impairment and transient paralysis. All patients had RRMS form 2 (Table 2). Among these patients, P1 had an very early onset of disease, at 6 years. She presented vision impairments, MRI demielinating supratentorial and infratentorial lesions. At that time, the case was interpreted as Acute Disseminated Encephalomyelitis (ADEM). The patient had no symptoms until the age of 14 years when the McDonald’s criteria for MS was met.

**Table 2:** Clinical data, where RR - relapse-remit.

Patient	Gender	Age onset	Onset symptoms	MS form
P1	F	6 years	visual impairment	RR
P2	M	14 years	right hand paresthesia, transient right hand paraly- sis, gait disturbances, type I diabetus mellitus ag- gravation	RR
P3	F	14 years	visual impairment, half body numbness	RR
P4	M	15 years	half body numbness	RR
P5	F	16 years	half body numbness, diplopia	RR
P6	F	17 years	visual impairment, optic nevritis	RR
P7	F	14 years	visual impairment, diplopia	RR
P8	F	17 years	half body numbness	RR
P9	F	17 years	numbness, balance disorders, gait disturbances, transient paralysis, peripheral facial paralysis	RR, Balo

We included in the dataset another interesting patient, P9, which developed a rare variant of MS, Balo’s concentric sclerosis. It was previously considered a severe form, but with advancements in neuroimaging techniques, it is now identified more frequently and may have a favorable prognosis. The concentric layout of alternating rings of hyper- and iso T2 signal intensity gives an *onion skin* appearance. These lesions can coexist with MS lesions.

Most patients initially presented with a high Expanded Disability Status Scale (EDSS) score, which decreased over time in several cases. For example, patient P2 had an initial EDSS score of 3.5, which improved to 0 during follow-up. In contrast, patient P3 showed fluctuations in EDSS over time, indicating variable disease activity. Overall, 7 out of 9 patients experienced relapses, ranging from 1 to 3 episodes, see Table 3. Patient 8 (P8) shows a large variation in the time intervals between scans, as some follow-up imaging was performed at other clinical centers.

**Table 3:** Clinical metrics.

Patient	Timepoint	EDSS	Relapses	Disease modyfing therapy?
P1			1	yes
	T1 - *t*_0_	3		
	T2 (*t*_0_ + 9.5 months)	1		
	T3 (*t*_0_ + 20.5 months)	1		
P2			0	yes
	T1 - *t*_0_	3.5		
	T2 (*t*_0_ + 3 months)	0		
	T3 (*t*_0_ + 12 months)	0		
	T4 (*t*_0_ + 17 months)	0		
	T5 (*t*_0_ + 31 months)	0		
	T6 (*t*_0_ + 42 months)	0		
P3			2	yes
	T1 - *t*_0_	2		
	T2 (*t*_0_ + 3 months)	2		
	T3 (*t*_0_ + 16 months)	1		
	T4 (*t*_0_ + 20 months)	2		
	T5 (*t*_0_ + 32 months)	1		
	T6 (*t*_0_ + 40 months)	2		
P4			2	yes
	T1 - *t*_0_	1		
	T2 (*t*_0_ + 3 months)	2		
	T3 (*t*_0_ + 8 months)	0		
	T4 (*t*_0_ + 12 months)	1		
P5			1	no
	T1	2		
P6			1	no
	T1	1		
P7			3	yes
	T1 - *t*_0_	1		
	T2 (*t*_0_ + 2 months)	1		
	T3 (*t*_0_ + 11 months)	1		
P8			0	yes
	T1 - *t*_0_	2		
	T2 (*t*_0_ + 2.5 years)	0		
	T3 (*t*_0_ + 5.1 years)	0		
P9			2	yes
	T1	1		

Each MRI scan underwent the same processing steps discussed in the following sections. Each patient was analysed, comprehensive clinical-metadata being described in Table 2. Finally, each MRI was labeled and a mask file was computed.

### Image acquisition

The MRI scanner used for patient examination was 3 T Siemens Magnetom Skyra. Each patient’s MRI scan consisted of a T1-MPRAGE (repetition time (TR) = 2000 ms, echo time (TE) = 2.27 ms, inversion time (TI) = 900 ms, flip angle (FA) = 8°, Slice Thickness = 1 mm, pixel sampling = 0.98 × 0.98 × 1 mm), a T2 (TR = 8870 ms, TE = 100 ms, FA = 150°, Slice Thickness = 3 mm, sampling = 0.47 × 0.47 × 3.9 mm) and a FLAIR image (TR = 9000 ms, TE = 81 ms, TI = 2500 ms, FA = 150°, Slice Thickness = 3 mm, sampling = 0.75 × 0.75 × 3.9 mm).

### Image processing

Each patient data was anonimized and defaced to ensure privacy. Next, we performed skull stripping on all MRI sequences using the SynthStrip^21,22^, a novel tool that produces accurate segmentation on a vast range of image types. The subsequent step consists of running N4-Bias Field correction from FSL^23^.

### Lesions delineation

The lesion delineation process was conducted by a junior rater and validated by two senior experts-a pediatric neurologist and a radiologist. Although lesion segmentation was performed primarily on FLAIR sequences, T1 and T2-weighted images were also analyzed to enhance accuracy and confidence.

Lesion delineation was performed using JIM 9.0 (https://xinapse.com/jim-9-software/) (Xinapse Systems Ltd., UK), a widely used tool for medical image analysis. The process involved loading the FLAIR image into JIM 9.0, utilizing the Contour feature to label the regions of interest (ROIs), and generating a corresponding lesion mask.

## Data Record

The dataset is available on figshare^20^ and it is structured as follows:

Each patient directory contains one or more timepoints. Within each timepoint directory, there are two subfolders:

- raw/ - Contains the original MRI scans, with the names FLAIR, T1 and T2.
- processed/ - Includes:
  - Skull stripped images: brain_FLAIR, brain_T1, brain_T2
  - Skull stipped masks: *mask_FLAIR, mask_T1, mask_T2*
  - N4 bias field-corrected images: *n4_brain_FLAIR, b4_T1_brain, n4_T2_brain*
  - Lesion segmentation mask for the FLAIR sequences: Consensus

All files are provided in NIFTI format.

## Technical Validation

To assess the quality and utility of the PediMS^20^ dataset, we performed inference using two different models: a previously developed model based on a state-of-the-art 2D UNet++^24^ architecture described in^9^, and an ensemble of three 3D U-Net models, referred to as LST-AI^25^. The UNet++ model, developed to evaluate generalizability^9^, was trained on the publicly available MSSEG-2016 test dataset^4^, which includes data from four different centers and imaging protocols. In contrast, LST-AI was trained on an in-house dataset.

### Evaluation metrics

For evaluation, we utilized the Dice Similarity Coefficient (DSC), the Jaccard Index (IoU), and the lesion-wise F1 score (F1) to assess segmentation performance.

$$\begin{array}{c}{DSC}=\frac{2{TP}}{2{TP}+{FP}+{FN}}\\ {IoU}=\frac{{TP}}{{TP}+{FP}+{FN}}\end{array}$$

$$F1{\rm{\_}}{lesionwise}=\frac{2{TP}{\rm{\_}}{lesionwise}}{2{TP}{\rm{\_}}{lesionwise}+{FP}{\rm{\_}}{lesionwise}+{FN}{\rm{\_}}{lesionwise}}$$

where

- TP represents the True Positives (the pixels accurately identified as multiple sclerosis lesions)
- TN – True Negatives (the pixels correctly identified as non-lesions)
- FP – False Positives (the pixels incorrectly predicted as MS lesions)
- FN – False Negatives (the pixels mistakenly classified as non-lesions).

### Dataset quality

State-of-the-art multiple sclerosis (MS) lesion segmentation methods on public datasets typically achieve a Dice score of approximately 0.67^9^. The 2D Unet++ model, trained on the MSSEG-2016 test dataset, achieved a Dice score of 0.541 and an F1 score of 0.586 when evaluated on the PediMS^20^ dataset, see Table 4. The LST-AI model, while obtaining a comparable Dice score of 0.536, demonstrated improved lesion detection with a higher F1 score of 0.714. These results support the quality of the PediMS^20^ dataset and its potential utility for advancing MS research and lesion segmentation methods. Nonetheless, the relatively low Dice scores indicate that there is still significant room for improvement in model performance on pediatric data.

**Table 4:** Inference results on the PediMS^20^ dataset.

Method	DSC score	IoU score	F1 score
2D UNet++^9^	0.541	0.374	0.586
LST-AI^21^	0.536	0.375	0.714

**Fig. 1:**
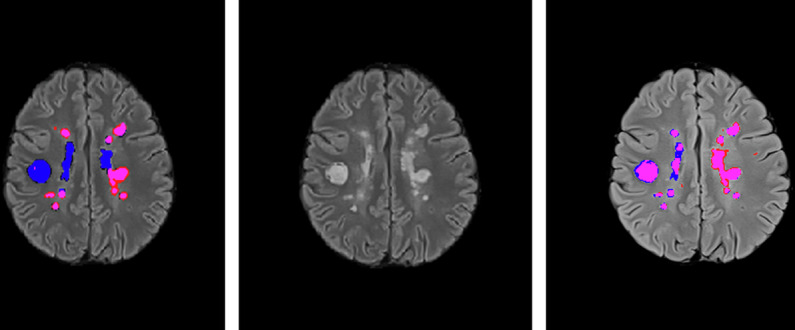
Patient 9 presenting Balo form of MS, where Magenta: True Positives, Red: False Positives, Blue: False Negatives (Ground Truth: Magenta + Blue, Predicted: Magenta + Red); Left image is the result of the 2D UNet++^9^ method, middle image is the original image and the right image is the result of LST-AI^25^.

Both models generalize well to new data, successfully identifying new lesions, though not entirely. For instance, in the case of patient P9, who presents Balo’s concentric sclerosis, the 2D UNet++ model struggled to detect the characteristic lesions, likely due to the absence of similar cases in the training data. It identified only two pixels in one of the three slices where the lesion is present. LST-AI identifies better the Balo lesion and also other lesions, but introduces more false positives (see Fig. 1).

**Fig. 2:**
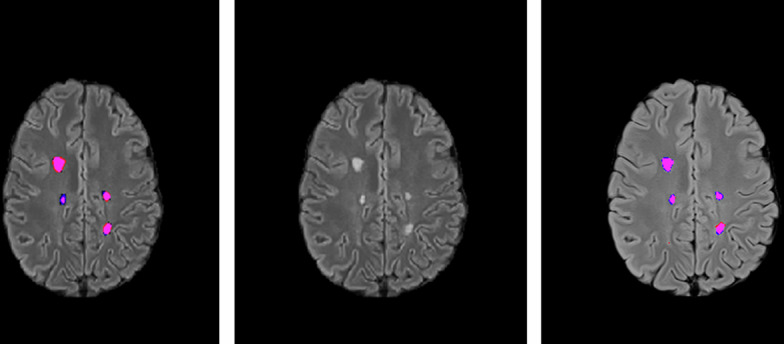
Patient 3, Timepoint 5 where Magenta: True Positives, Red: False Positives, Blue: False Negatives (Ground Truth: Magenta + Blue, Predicted: Magenta + Red); Left image is the result of the 2D UNet++^9^ method, middle image is the original image and the right image is the result of LST-AI^25^.

Common lesions in multiple sclerosis are almost perfectly detected by both models, once again demonstrating the high quality of the dataset. Figure 2, from Patient 3 at timepoint 5, shows a slice in which all lesions are identified.

Although both methods yield relatively low Dice coefficients and IoU scores, this highlights the need for more data to enable better generalization of the models. The proposed dataset introduces a category of patients not previously included in publicly available datasets. PediMS^20^ is a pediatric dataset that features patients with more numerous and larger lesions, and it also includes a case with a rare form of MS. The accompanying clinical data and metrics are provided to support further research and development of new methods that can leverage this information.

Advances in deep learning have produced a wide range of state-of-the-art segmentation methods. However, the current challenge is to develop models that can recognize different types of lesions, which underscores the need for new and diverse datasets.

To develop robust models capable of recognizing diverse lesion types, it is essential to include a wide range of MS lesion types and patient variations. The proposed dataset contributes to this effort by facilitating the creation of more accurate segmentation models and supporting future federated learning approaches.

## Data Availability

The code is available on Github: https://github.com/marypopa/PediMS.
